# Association between depressive symptoms and diagnosis of diabetes and its complications: A network analysis in electronic health records

**DOI:** 10.3389/fpsyt.2022.966758

**Published:** 2022-09-23

**Authors:** Cheng Wan, Wei Feng, Renyi Ma, Hui Ma, Junjie Wang, Ruochen Huang, Xin Zhang, Mang Jing, Hao Yang, Haoran Yu, Yun Liu

**Affiliations:** ^1^Department of Medical Informatics, School of Biomedical Engineering and Informatics, Nanjing Medical University, Nanjing, China; ^2^Department of Medical Psychology, Nanjing Brain Hospital, Nanjing Medical University, Nanjing, China; ^3^Department of Information, The First Affiliated Hospital, Nanjing Medical University, Nanjing, China

**Keywords:** type 2 diabetes mellitus, depressive symptoms, natural language processing, network analysis, diabetes complication

## Abstract

**Objectives:**

Diabetes and its complications are commonly associated with depressive symptoms, and few studies have investigated the diagnosis effect of depressive symptoms in patients with diabetes. The present study used a network-based approach to explore the association between depressive symptoms, which are annotated from electronic health record (EHR) notes by a deep learning model, and the diagnosis of type 2 diabetes mellitus (T2DM) and its complications.

**Methods:**

In this study, we used anonymous admission notes of 52,139 inpatients diagnosed with T2DM at the first affiliated hospital of Nanjing Medical University from 2008 to 2016 as input for a symptom annotation model named T5-depression based on transformer architecture which helps to annotate depressive symptoms from present illness. We measured the performance of the model by using the F1 score and the area under the receiver operating characteristic curve (AUROC). We constructed networks of depressive symptoms to examine the connectivity of these networks in patients diagnosed with T2DM, including those with certain complications.

**Results:**

The T5-depression model achieved the best performance with an F1-score of 91.71 and an AUROC of 96.25 compared with the benchmark models. The connectivity of depressive symptoms in patients diagnosed with T2DM (*p* = 0.025) and hypertension (*p* = 0.013) showed a statistically significant increase 2 years after the diagnosis, which is consistent with the number of patients diagnosed with depression.

**Conclusion:**

The T5-depression model proposed in this study can effectively annotate depressive symptoms in EHR notes. The connectivity of annotated depressive symptoms is associated with the diagnosis of T2DM and hypertension. The changes in the network of depressive symptoms generated by the T5-depression model could be used as an indicator for screening depression.

## 1. Introduction

Depression is two times more common in patients with diabetes in the general population (1). Diabetic patients with depression have difficulty controlling their glycemic index (2), have a higher risk of dementia (3), and experience higher health care costs (4), especially in older adults (5). Depression has been reported to promote high risks of poor glucose control, comorbidities, and mortality in diabetic patients with depression (5, 6).

The duration of diabetes is associated with depression (7). A trial involving people who were newly diagnosed with type 2 diabetes mellitus (T2DM) found that the prevalence of depressive symptoms increased at least 1 year post diagnosis (8). Depressive symptoms are also associated with the presence of complications of diabetes, including cardiovascular disease, cerebrovascular disease, and neuropathy (9–12). This sentence highlights that the diagnosis events of complications, but the previous sentence describes about the relation between complications and depressive symptoms.

Depressive symptoms were estimated mostly using sum scores based on reported symptoms from screening tools (8, 13). The widely used validated tools for depression screening include the Patient Health Questionnaire-9 (PHQ-9), the Center for Epidemiologic Studies Depression Scale, and the World Health Organization-5 Well Being Index (WHO-5) (13, 14). Although treatment guidelines for patients with diabetes recommend regular depression screening (15), the rate of depression screening for such patients is low (16–18). However, owing to the heterogeneity of depression, the sum scores used in screening tools ignore the interactions among depressive symptoms when estimating the severity of symptoms (19). The low rate of depression screening and a lack of information on symptom interaction limit the identification of depressive symptoms and the understanding of their correlations in patients with diabetes.

The development of natural language processing (NLP) models and data mining in large-scale clinical real-world datasets of electronic health records (EHRs) has promoted screening for depression in patients with diabetes (18), especially screening based on admission notes. These notes contain a history of past illness, present illness, allergy, and birth information (20). Present illness includes the patient's main complaints, narratives of symptoms, and progress of treatment during their time in the hospital. During their hospital stay, patients will be checked for their status of spirit, sleep quality, and appetite, among others. Some researchers successfully used NLP tools to extract symptom data from unstructured free-text clinical documents in EHR. For example, Geraci et al. (21) extracted data from clinical notes through an NLP technique to predict the diagnosis of depression. Patel et al. (22) investigated the associations of depressive symptoms with clinical outcomes. These research studies provide opportunities for identifying depression-related symptoms and computer-aided diagnosis (23, 24).

In contrast to screening tools, network connectivity of symptoms focuses on estimating the associations among symptoms. The symptom–symptom interactions are used to form a network structure in the network analysis (25). Based on the network structure of depressive symptoms, the associations between symptoms and disease can be estimated from a part-whole perspective (26). Increases in network connectivity are associated with the severity of depression and persistent depressive symptoms (27, 28). Therefore, network analysis has recently been used as an alternative approach to assessing the severity of depressive symptoms.

In the present study, a model for classification and analysis of depression-related symptoms was proposed to directly identify depression symptoms from the EHRs of inpatients. Data were collected from an observational clinical dataset of inpatients at the First Affiliated Hospital of Nanjing Medical University over 8 years. The model integrated a transformer model with network analysis to facilitate an increased screening rate while retaining symptom interactions. The study examined depressive symptoms in patients with four complications of diabetes. The overall and local connectivities of the resulting networks were compared with the diagnosis of depression.

## 2. Methods

### 2.1. Study design and setting

The present study obtained medical record data from the Observational Medical Outcomes Partnership Common Data Model (OMOP CDM) at the First Affiliated Hospital of Nanjing Medical University (29). In this dataset, 61,471 inpatients with valid present-illness notes were selected from among the 148,624 patients in the CDM who had been diagnosed with T2DM. We included data on patients' age at T2DM diagnosis, sex, diagnosis of depression (ICD-10 codes F32–F33), and diagnosis of complications: hypertension (ICD-10 code I10), ischemic heart disease (ICD-10 codes I20–I25), and cerebrovascular disease (ICD-10 codes I60–I69). The date of diagnosis of T2DM was defined as the earliest date of hemoglobin A1C ≥ 48 mmol/mol, or 6.5%, or use of insulin, or oral hypoglycemic drugs, or the first recorded diagnosis of T2DM. The date of diagnosis of complications was defined as the first recorded diagnosis of the complications. Ethical approval for the study was received from the Ethics Committee of the First Affiliated Hospital, Nanjing Medical University, Jiangsu, China.

We identified 52,139 inpatients who were first diagnosed with T2DM between 2008 and 2016 from a total of 61,471 patients. The complications (ischemic heart disease, hypertension, or cerebrovascular disease) of T2DM were confirmed after the diagnosis of T2DM had been made. To examine the difference in depressive symptoms before and after the diagnosis of T2DM or complications, a filtering rule (admission pairs, APs) was defined: patients were required to have admission records before and after the date of disease diagnosis during 2 years. For example, “patients have AP for hypertension” meant that patients had admission records 2 years before and after the date of diagnosis of hypertension. Eligible patients had AP for T2DM or its complications. Once the eligibility criteria had been met, 8,885 patients with T2DM, 1,357 patients with ischemic heart disease, 2,619 patients with hypertension, and 1,693 patients with cerebrovascular disease were selected for analysis. The procedure of patient selection is shown in Supplementary Figure S1. The year when patients were first diagnosed with T2DM, hypertension, ischemic heart disease, or cerebrovascular disease was coded as 0. Admissions in the preceding 2 years were coded as –2 and successive admissions as 2.

### 2.2. Depressive symptoms annotation

#### 2.2.1. Processing of labeled datasets

To limit the cost of labeling, we randomly selected 10% of (15,615) notes from 156,156 admission notes of all inpatients (61,471 patients) to build and test the annotation model. Repeated notes of present-illness were excluded. Once the eligibility criteria had been met, 13,880 valid records of present-illness (dataset I) were labeled. To train, validate, and test the model, this dataset was split according to a ratio of 8:1:1. In addition, to internally evaluate the performance of the model, we randomly selected 4,658 admission notes (10% of 46,583 notes) as dataset II. These notes were extracted from the samples obtained in the previous patient selection process (see the “Study design and setting” section). Three annotators completed the labeling process of both datasets under the training of a clinical expert. A diagram illustrating this procedure is shown in Supplementary Figure S2.

We summarized depressive symptoms according to items described in previous research (30) and two commonly used screening tools namely, PHQ-9 (31) and WHO-5 (32). We included increased and decreased weights as symptoms because they were reported to have an effect on increasing the risk of depression in patients with T2DM (30). Another nine unique depressive symptoms were selected from PHQ-9 and WHO-5 scales, including feeling tired, difficulty in sleeping, a decrease in appetite, moving slowly, feeling irritable, a decline in memory or attention, feeling dispirited, depressed, or anxious, and feeling suicidal. The relationships of items in the screening tools and symptoms are shown in Supplementary Table S2. Antonyms were used for items in WHO-5 because these items are both positive expressions compared to PHQ-9. A total of 11 candidate depressive symptoms were evaluated by clinical experts. The synonyms of candidate depressive symptoms were also provided by clinical experts and were used in labeling (Supplementary Table S3).

Descriptive statistics for the manual labeling of datasets I and II are shown in Supplementary Table S4. Symptoms that occurred fewer than 10 times in any dataset were excluded. Finally, nine symptoms (feeling tired, difficulty in sleeping, a decrease in appetite, moving slowly, feeling irritable, a decline in memory or attention, a decrease in weight, an increase in weight, and feeling dispirited) were selected to build the model.

#### 2.2.2. Annotation model development and evaluation

**Figure 1:**
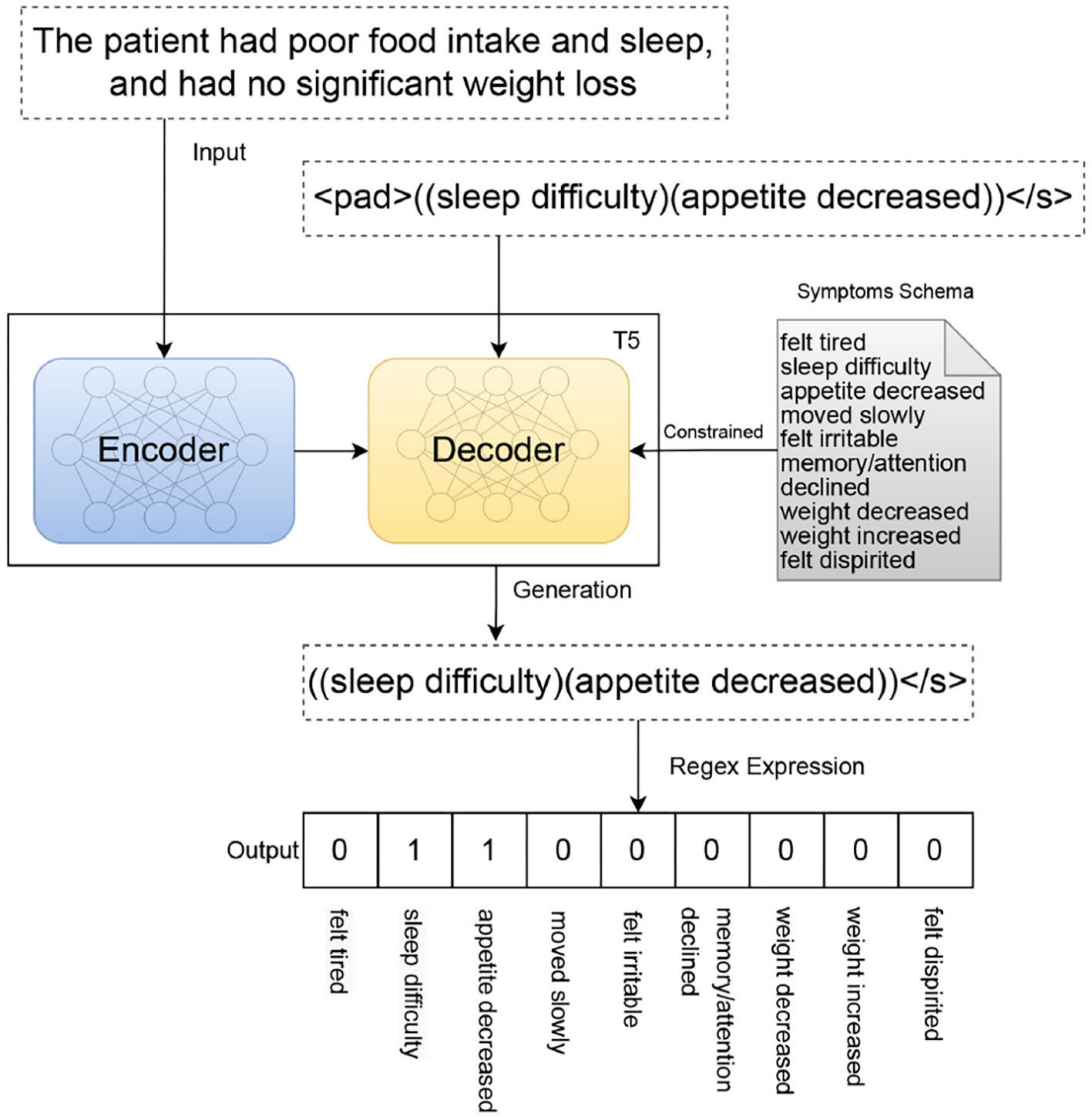
Architecture of the T5-depression model with formatted input and output.

To annotate the depressive symptoms, we built a model named T5-depression based on a transformer model, Transfer Text-to-Text Transformer (T5 model) (33) model, with a sequence-to-structure paradigm. The architecture of T5-depression is shown in Figure 1. This model contains three parts: two multi-head attention modules (the encoder and decoder modules) and an auto rule-based result conversion module. The encoder module changes the input sentence *x* into a contextualized representation. The decoder module predicts the output sentence *y* on a token-by-token basis in the structured sequence. A constrained decoding algorithm for the symptom schema is injected during inference. The probability of the output is given in Equation 1, where *T* represents the size of the output and *y*_*i*_ represents each step of the output:


(1) $$\begin{array}{l} P\left(y|x\right)=\overset{T}{\underset{i=1}{\prod }}p\left(y_{i}|y_{1},...,y_{i-1},x\right) \end{array}$$

In the present study, each present illness note was regarded as the input, and all the related symptoms were formatted as the output. An example of formatted input and output is shown in Figure 1. To support the Chinese language, we replaced the original pretrained model T5-base (33) with T5-Mengzi (34). Among the super-parameters used in T5-depression model, the learning rate was set to 5e-5, training epochs to 25, and batch size to 16. Other super-parameters were set as same as in Text2event.

We compared the performance of our model with a rule-based model, the Bidirectional Encoder Representations from Transformers (Bert) model (35), and the Roberta model (36) as benchmarks. The rule-based model was implemented using regular expressions derived from synonyms of each symptom in Supplementary Table S1. Roberta (36) was pretrained with Chinese-Roberta-wwm-ext (37) and Bert (35) was pretrained with Bert-base-Chinese. Bert is a contextualized word representation model that has been applied successfully to several tasks in the medical domain (38, 39). In the present study, we used the same number of epochs, batch size, and learning rate for Roberta and Bert. The implementation of the benchmark model was based on the Transformers package (40). All code for this study has been uploaded to https://github.com/inseptember/T5-depression.

To evaluate the performance of the models, precision, recall, F1-score, HammingLoss (41), and area under the receiver operating characteristic curve (AUROC) were used as metrics. Definitions of these metrics are given in Eqs. S1–S4 (Supplementary material). Precision is the ability to identify true positive samples among all positive prediction results. Recall is the ability to identify true positive samples among all positive results. F1 is the harmonic mean of the precision and recall. HammingLoss is the minimum number of substitutions required to change one sequence to another. This study indicates the fraction of symptoms that were incorrectly predicted. Owing to the unbalanced symptoms in our dataset (Supplementary Table S4), micro-averaging was performed on all symptoms. To prevent incorrect prediction of present illness notes, a model with higher recall and F1 is preferred to one with higher precision, AUROC, or HammingLoss. All metrics were calculated using the scikit-learn Python package (42).

Finally, T5-depression was used to process all present illness notes. The depressive symptoms, annotated from present illness notes within the range of admissions, were marked as binary values (1 for yes and 0 or no), depending on whether the notes contained the symptoms or not.

### 2.3. Statistical analysis

In depressive symptom networks, nodes represent symptoms and edges represent the associations between symptoms (26). We used the Ising models with the extended Bayesian information criterion from the R package *bootnet* (43) to construct and estimate the centrality of depressive symptoms before and after the diagnosis of each T2DM complication. We set the hyper-parameter tuning to 0 to estimate more connections (28). The weighted networks were illustrated using the R package *qgraph* (44).

Statistical assessment of the differences in overall connectivity of networks was performed using the R package *NetworkComparisonTest* (NCT) (27, 45). NCT is a two-tailed permutation-based hypothesis test to assess the difference between two groups (in our case, before and after the diagnosis of a disease or complication). The R package was run 1,000 times in our study to observe the differences in overall connectivity between the networks. In addition, differences in the importance of nodes were measured by node strength, closeness, and betweenness (44). Strength describes the degree to which a node is connected to other nodes. Closeness measures how close a node is to other nodes. Betweenness assesses the degree to which a node lies on the shortest path between nodes (46). In the present study, node strength was used as a local connectivity index. Furthermore, χ^2^ test was conducted to determine depression associated difference for each complication. We compared the results from this test to the overall difference from the NCT package.

## 3. Results

### 3.1. Participants

A total of four cohorts with a diagnosis of T2DM or its complications (hypertension, ischemic heart disease, and cerebrovascular disease) were selected for this study. Descriptive statistics are shown in Table 1. The mean ages of participants in these four cohorts were 60.36 ± 15.58, 62.07 ± 13.03, 66.06 ± 14.15, and 66.52 ± 13.92, respectively. More than 59% of patients were men. Of the 8,885 patients in the T2DM cohort, 60 patients developed depression after diagnosis of T2DM within 2 years (*p* < 0.001). Of the 2,619 T2DM patients with hypertension, 24 developed depression after diagnosis with hypertension within 2 years (*p* < 0.01). There were no statistically significant differences in the diagnosis of depression after diagnosis with ischemic heart disease or cerebrovascular disease in patients with T2DM within 2 years.

**Table 1:** Descriptive statistics for each diseases in these windows of years.

	**T2DM**			**Hypertension**			**Ischaemic heart disease**			**Cerebrovascular disease**		
	**(–2,0]**	**(0,2]**	***p***	**(–2,0]**	**(0,2]**	***p***	**(–2,0]**	**(0,2]**	***p***	**(–2,0]**	**(0,2]**	***p***
Total, n	8885			2619			1357			1693		
Age, Mean (SD)	60.36 (15.58)			62.07 (13.03)			66.06 (14.15)			66.52 (13.92)		
Sex, n (%)												
Men	5242 (59.00)		0.0000^***^	1643 (62.73)		0.0000^***^	911 (67.13)		0.0000^***^	1051 (62.08)		0.0000^***^
Women	3643 (41.00)			976 (37.27)			446 (32.87)			642 (37.92)		
Depression, n (%)	81 (0.91)	141 (1.59)	0.0001^***^	21 (0.80)	45 (1.72)	0.0044^**^	40 (2.95)	31 (2.28)	0.3360	55 (3.25)	69 (4.08)	0.2343
Depressive Symptoms, n (%)												
Feeling tired	1,569 (17.66)	1,797 (20.23)	0.0000^***^	555 (21.19)	589 (22.49)	0.2698	276 (20.34)	287 (21.15)	0.6359	531 (31.36)	498 (29.42)	0.2318
Difficulty in sleeping	1,225 (13.79)	1,396 (15.71)	0.0003^***^	443 (16.91)	479 (18.29)	0.2041	261 (19.23)	278 (20.49)	0.4414	345 (20.38)	347 (20.50)	0.9660
A decrease in appetite	1487 (16.74)	1,735 (19.53)	0.0000^***^	476 (18.17)	554 (21.15)	0.0074^**^	235 (17.32)	277 (20.41)	0.0443^*^	341 (20.14)	335 (19.79)	0.8298
Moving slowly	99 (1.11)	155 (1.74)	0.0005^***^	43 (1.64)	54 (2.06)	0.3054	14 (1.03)	19 (1.40)	0.4836	55 (3.25)	73 (4.31)	0.1256
Feeling irritable	47 (0.53)	61 (0.69)	0.2096	18 (0.69)	22 (0.84)	0.6340	12 (0.88)	9 (0.66)	0.6613	21 (1.24)	26 (1.54)	0.5568
A decline in Memory/attention	60 (0.68)	89 (1.00)	0.0212^*^	25 (0.95)	36 (1.37)	0.1978	12 (0.88)	15 (1.11)	0.6989	48 (2.84)	59 (3.48)	0.3259
A decrease in Weight	1051 (11.83)	1,138 (12.81)	0.0496^*^	417 (15.92)	331 (12.64)	0.0008^**^	118 (8.70)	137 (10.10)	0.2363	182 (10.75)	158 (9.33)	0.1885
An increase in Weight	99 (1.11)	179 (2.01)	0.0000^***^	36 (1.37)	55 (2.10)	0.0570	15 (1.11)	17 (1.25)	0.8589	18 (1.06)	23 (1.36)	0.5297
Feeling dispirited	358 (4.03)	472 (5.31)	0.0001^***^	146 (5.57)	160 (6.11)	0.4438	77 (5.67)	69 (5.08)	0.5515	140 (8.27)	148 (8.74)	0.6663

For age, standard deviation is in parentheses, mean value is outside. For sex and depressive symptoms, percentage value is in parentheses, amount value is outside.

(–2, 0], within 2 years before diagnosed date of each disease; (0, 2], within 2 years after diagnosed date of each disease.

p, p-values from χ2-test; ^*^ <0.05; ^**^ <0.01; ^***^ <0.001.

### 3.2. Performance and annotation results of the T5-depression model

Table 2 reports the performance of the models for the annotation of depressive symptoms. Compared with other models, the T5-depression model achieved the best performance, with a micro-average F1 of 91.71% in dataset I and 95.53% in dataset II. The rule-based approach had the highest precision value of 95.39% and the lowest recall value of 79.23% in dataset I. The Roberta model achieved the best HammingLoss of 0.0027 in dataset I. The Bert model did not outperform other models in both datasets. We chose the T5-depression model as the auto-annotation model for the present-illness notes of patients.

**Table 2:** Metrics for different models on Dataset I and II.

	**Pretrained model**	**Datasets**	**AUROC**	**Hamming loss**	**P**	**R**	**F1**
Rule-based	-	Dataset I - Train	89.74	0.0226	95.99	79.83	87.17
		Dataset I - Test	89.41	0.0242	95.39	79.23	86.57
		Dataset I - Validation	89.41	0.0243	96.57	79.14	86.99
		Dataset II	82.06	0.0263	92.91	64.45	76.11
Roberta	hfl/chinese-roberta-wwm-ext (37)	Dataset I - Train	93.78	0.012	94.8	89.39	92.02
		Dataset I - Test	93.21	0.0027	90.65	87.81	89.21
		Dataset I - Validation	92.98	0.0028	90.59	85.88	88.17
		Dataset II	89.32	0.0154	91.64	84.04	87.68
Bert	bert-base-chinese^*a*^	Dataset I - Train	90.51	0.0173	91.72	85.39	88.44
		Dataset I - Test	88.46	0.003	92.25	83	87.38
		Dataset I - Validation	91.2	0.0031	91.12	83.07	86.91
		Dataset II	88.38	0.0156	92.49	82.69	87.31
T5-depression	Langboat/mengzi-t5-base (34)	Dataset I - Train	99.3	0.0021	99.16	98.7	98.93
		Dataset I - Test	96.25	0.0166	89.84	93.65	91.71
		Dataset I - Validation	95.37	0.0173	91.43	91.72	91.58
		Dataset II	97.47	0.0058	95.83	95.23	95.53

P, Precision; R, Recall; F1, F-scores.

^*a*^: https://huggingface.co/bert-base-chinese.

According to annotation with the T5-depression model, the top three depressive symptoms in all cohorts were feeling tired, difficulty in sleeping, and a decrease in appetite (Table 1). The percentage of each depressive symptom increased significantly in the 2 years after diagnosis in the T2DM cohort, except for feeling irritable. Symptoms of a decrease in appetite (*p* < 0.01) and a decrease in weight (*p* < 0.01) showed a significant increase in the 2 years after diagnosis in the T2DM and hypertension cohort. Only a decrease in appetite (*p* < 0.05) showed a significant increase in the ischemic heart disease cohort. No change in symptoms was found in the cerebrovascular disease cohort.

### 3.3. Network analysis

**Figure 2:**
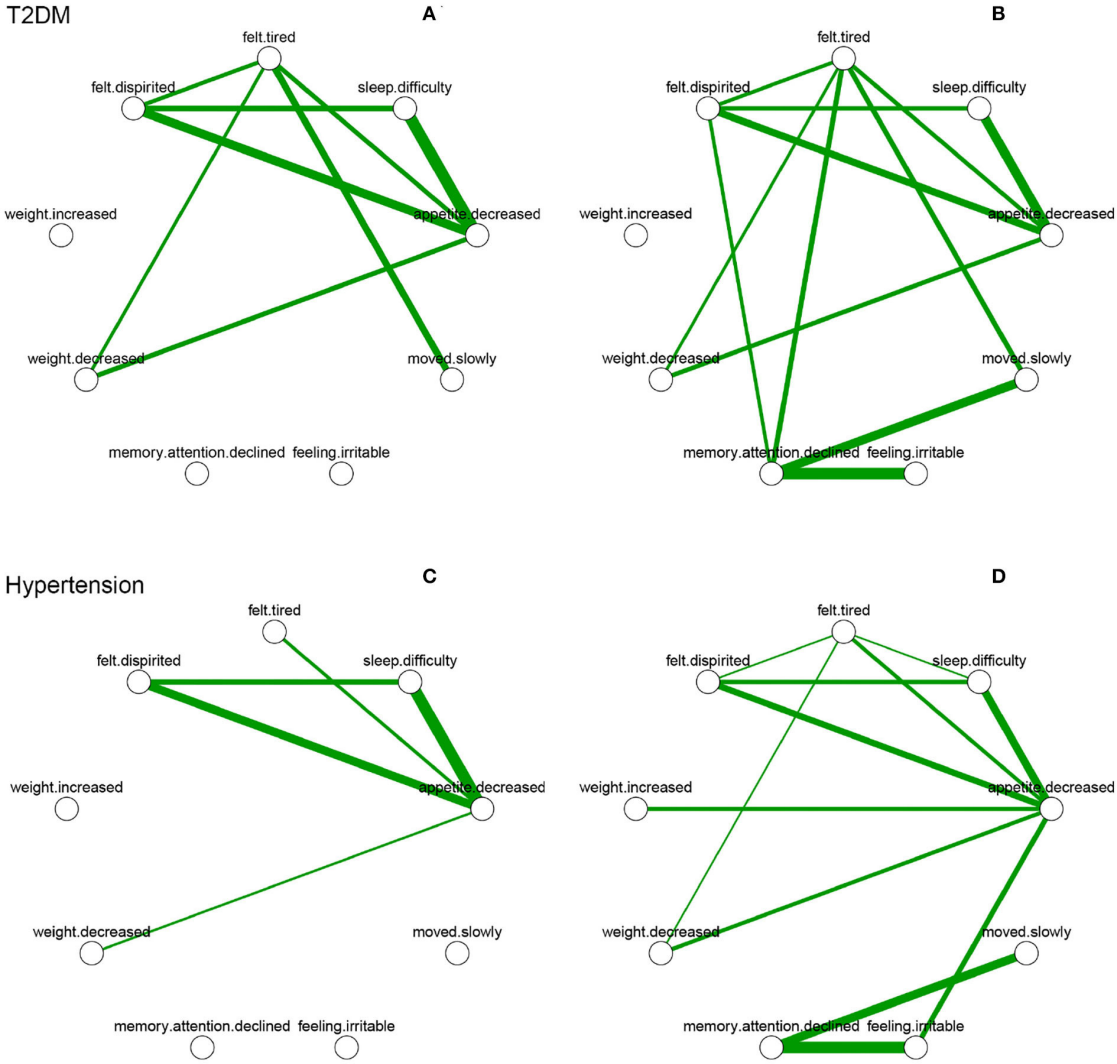
Depressive symptom networks within 2 years before and after the diagnosis of T2DM and hypertension. Green lines represent positive association, and thicker lines represent stronger association. **(A)** is within 2 years before diagnosed date of T2DM. **(B)** is within 2 years after diagnosed date of T2DM. **(C)** is within 2 years before diagnosed date of hypertension. **(D)** is within 2 years after diagnosed date of hypertension.

A comparison of overall network connectivity before and after diagnosis (with T2DM, hypertension, ischemic heart disease, and cerebrovascular disease) is presented in Table 3. After diagnosis of diabetes (*p* = 0.025) and hypertension (*p* = 0.013) in patients, the overall connectivity of the symptom networks increased significantly during the 2 years. Due to consistent increase in overall connectivity, the number of patients with T2DM and hypertension diagnosed with depression also increased. With regard to symptoms, strong positive connections between a decline in memory/attention and feeling irritable were found after diagnosis of T2DM and hypertension (Figure 2). The strength of symptoms was measured as local connectivity (Supplementary Figure S2). The symptom of a decrease in appetite remained high and stable both before and after diagnosis. Weight-related symptoms had relatively lower values.

**Table 3:** Comparison between network connectivity for each disease.

	**Overall connectivity**			**Depression diagnosis**		
	**(–2,0]**	**(0,2]**	***p*-value**	**(–2,0]**	**(0,2]**	**χ^2^**
T2DM	7.54	13.79	0.0250^*^	81	141	0.0001^***^
Hypertension	4.60	13.47	0.0130^*^	21	45	0.0044^**^
Ischaemic Heart Disease	3.91	5.37	0.3250	40	31	0.3360
Cerebrovascular Disease	7.21	8.83	0.5860	55	69	0.2343

*P*-values in overall connectivity are computed from the Network Comparison Test.

(–2, 0], within 2 years before diagnosed date of each disease; (0, 2], within 2 years after diagnosed date of each disease.

^*^ <0.05; ^**^ <0.01; ^***^ <0.001.

## 4. Discussion

In the present study, we compared depressive symptoms from the EHR notes of patients in the 2 years before and after the diagnosis of T2DM and complications (hypertension, ischemic heart disease, and cerebrovascular disease). To annotate depressive symptoms in EHR notes of patients with T2DM, we applied the T5-depression model to automatically label depressive symptoms. We used network analysis to examine the differences among depressive symptom networks. The diagnoses of T2DM and hypertension were associated with increased overall connectivity of the symptom network.

The T5-depression model was built using a sequence-to-structure paradigm. Classic methods used for symptom annotation include rule-based approaches and form part of pipelines including MedLEE (47), ClinREAD (48), and MTERMS (49). The rule-based methods mostly extract symptoms through keyword retrieval, which ignores the context semantics of each symptom. Recently, Bert (35) was applied to various clinical tasks (50–52). Through a multi-head attention mechanism, Bert-related models can obtain context features of each symptom. Moreover, methods used for annotation tasks train a classifier on the last layer of the model. However, this requires much effort when applied to medical notes with fine-grained token-level annotation. The sequence-to-structure paradigm model can generate all targets in one step and show competitive performance using only record-level annotations. This mechanism can reduce much of the effort involved in the annotation procedure and has generalizability (53).

In the present study, the T5-depression model achieved promising performance on our dataset. The architecture of T5-depression was designed to translate the input text to an augmented readable text, which makes the annotation procedure easier and enables the handling of labels of different sizes with less modification than classification models with one-hot labels. T5-depression can make full use of semantic information of labels by relative positional embeddings and use of an autoregressive decoder, unlike Bert-related models. Owing to the smaller number of annotations of certain symptoms, including feeling irritable and a decline in memory or attention (Supplementary Table S4), the rule-based model achieved the highest precision; however, it had a lower recall value than the T5-depression model. Through keyword searching, the rule-based approach could identify the exact same symptoms as labeled in notes but failed to identify symptoms in other formats. The T5-depression model showed poor performance related to symptoms including moving slowly (F1: 70.59) and feeling irritable (F1: 70.27) because these two symptoms had a more varied expression in the Chinese population and were reported in limited numbers compared with other symptoms in our dataset. The T5-depression model still achieved the best overall performance compared with regular rule-based annotation methods. Thus, the model could be a reliable tool for identifying depressive symptoms in EHR of patients with T2DM, thereby saving time and effort on the part of medical experts.

This study showed a significant increase in the overall connectivity of depressive symptoms during the 2 years after diagnosis of T2DM and hypertension. Persistent depressive symptoms have been reported in the early stages of diabetes (8), and awareness of hypertension indicates a higher prevalence of depressive symptoms (54). It has been reported that persistent depressive symptoms are associated with stronger network connectivity (27), and the change in overall connectivity in this study was consistent with the diagnosis of depression in patients with T2DM and hypertension. Although some studies have reported that cerebrovascular disease has stronger associations with depressive symptoms in patients with diabetes (11, 55), the short-term prevalence of depression was unchanged (56), which is consistent with our results. The network connectivity of depressive symptoms in the early stages of T2DM could be an indicator for further monitoring of depression while current method for screening depression in patients with diabetes is insufficient. Although a study has suggested that the development of T2DM might not induce depressive symptoms (2), the increase in frequency of depressive symptoms after the diagnosis of T2DM illustrates the importance of mining EHR notes for psychiatric research.

High centrality of symptoms in the network could be considered as important parts in maintaining the mental disorders (25). We used the strength of nodes to measure local connectivity in this study. For patients diagnosed with T2DM and complications including hypertension, the centrality of the symptom of a decline in memory or attention ranked top among all depressive symptoms over 2 years. Depression is associated with concurrent cognitive decline (57) in patients with diabetes. Nonpharmacological treatments for behavioral and psychological symptoms of dementia have been shown to result in improvements in depression (58). Some studies also reported that anti-dementia drugs have antidepressant-like effects (59, 60) in animal models. Treatment of cognitive symptoms is thus a potential approach for the prevention of depression in patients with diabetes.

## 5. Limitations

Our study had some limitations. First, we included admissions only 2 years before and after the initial diagnosis; this time period might not be enough given that this was a study of chronic disease, in which some patients may have developed before their first admission or diagnosis in the hospital. Second, the annotation model for present illness in EHR notes has the potential to improve precision on some limited symptoms. In the current study, the semantics of tags helped to improve the performance of the model. In the future, we could inject a detailed description of each symptom obtained from multi-modal resources to the model and fuse vital indicators with symptom descriptions in the model. Third, the selected symptoms in this study might not cover the whole range of depressive symptoms compared with other screening tools like CES-D. Some associations of symptoms could therefore have been missed. More screening tools should be examined in the future to include more symptoms.

## 6. Conclusion

In the present study, depressive symptoms were annotated effectively from EHR notes by the T5-depression model, and network analysis was used to examine the effects of the diagnosis of T2DM and complications on depression. The model achieved acceptable performance on the annotation of depressive symptoms in all datasets, and the connectivity of depressive symptom networks was shown to be associated with the diagnosis of T2DM and hypertension during the past 2 years. In future research, the transition of symptoms during the course of T2DM should be examined, and more symptoms should be included in the model to estimate the relationships with other vital indicators.

## Data availability statement

The datasets presented in this article are not readily available because data privacy and security requirements. Requests to access the datasets should be directed to YL, liuyun@njmu.edu.cn.

## Ethics statement

The studies involving human participants were reviewed and approved by the Ethics Committee of the First Affiliated Hospital, Nanjing Medical University (2020-SR-163). Written informed consent for participation was not required for this study in accordance with the national legislation and the institutional requirements.

## Author contributions

WF and CW contributed to conception and design of the study and performed the statistical analysis and wrote the first draft of the manuscript. HM and XZ organized the database. JW and RH prepared the figures in manuscript. HYa and HYu conducted the process of data collection. YL supervised the research activity planning and execution. RM and MJ annotated the datasets and participated in revising the manuscript. All authors contributed to manuscript revision, read, and approved the submitted version.

## Funding

This work was supported by the Industry Prospecting and Common Key Technology Key Projects of Jiangsu Province Science and Technology Department (Grant no. BE2020721), the National Key Research & Development Plan of Ministry of Science and Technology of China (Grant nos. 2018YFC1314900 and 2018YFC1314901), the Industrial and Information Industry Transformation and Upgrading Special Fund of Jiangsu Province in 2021 [Grant no. (2021)92], the Industrial and Information Industry Transformation and Upgrading Special Fund of Jiangsu Province in 2018 [Grant no. (2019)55], the Key Project of Smart Jiangsu in 2021 [Grant no. (2021)9], the Key Project of Smart Jiangsu in 2020 [Grant no. (2021)1], and Jiangsu Province Engineering Research Center of Big Data Application in Chronic Disease and Intelligent Health Service [Grant no. (2020)1460].

## Conflict of interest

The authors declare that the research was conducted in the absence of any commercial or financial relationships that could be construed as a potential conflict of interest.

## Publisher's note

All claims expressed in this article are solely those of the authors and do not necessarily represent those of their affiliated organizations, or those of the publisher, the editors and the reviewers. Any product that may be evaluated in this article, or claim that may be made by its manufacturer, is not guaranteed or endorsed by the publisher.
