# Supplementary material for: Association between depressive symptoms and diagnosis of diabetes and its complications: A network analysis in electronic health records
Source: Front Psychiatry. 2022 Sep 23;13:966758. doi: 10.3389/fpsyt.2022.966758 (PMC9543719; doi:10.3389/fpsyt.2022.966758)
Supplement: Supplementary file 1 [file Data_Sheet_1.PDF]

Supplementary Material

1 SUPPLEMENTARY DATA

1.1 Equations

$$Precision = \frac{TP}{(TP + FP)} \tag{S1}$$

$$Recall = \frac{TP}{(TP + FN)} \tag{S2}$$

$$F_1 = \frac{2 \times Precision \times Recall}{(Precision + Recall)} \tag{S3}$$

$$HammingLoss = \frac{1}{nm} \sum_{i=1}^{n} \sum_{j=1}^{m} 1(y_j^{(i)} \neq \hat{y}_j^{(i)}) \tag{S4}$$

True positive (TP) means that the symptom exists in the notes used as input to the model and the classifier results output by the model are correct. FP means false positive (i.e., the symptom does not exist in the input notes and the model has predicted wrongly). FN means false negative (i.e., the symptom exists in the notes but the model has predicted wrongly). m is the label size, $\hat{y}$ is the predicted value, and $y_j^{(i)}$ is the j-th label of the i-th sample.

1.2 Figures

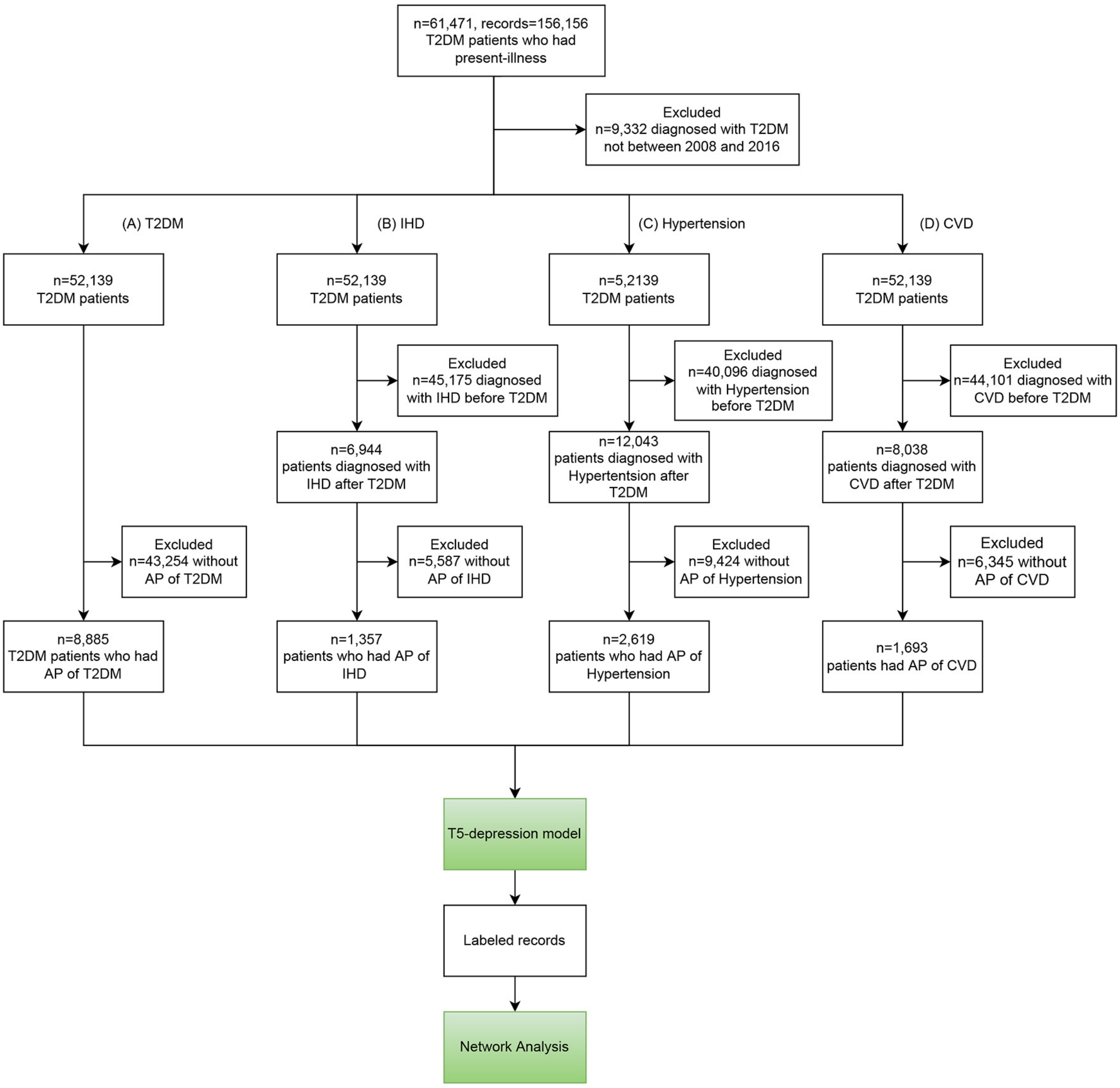

Figure S1. Flowchart of patient selection. Abbreviations: T2DM, type 2 diabetes mellitus; IHD, ischemic heart disease, CVD, cerebrovascular disease

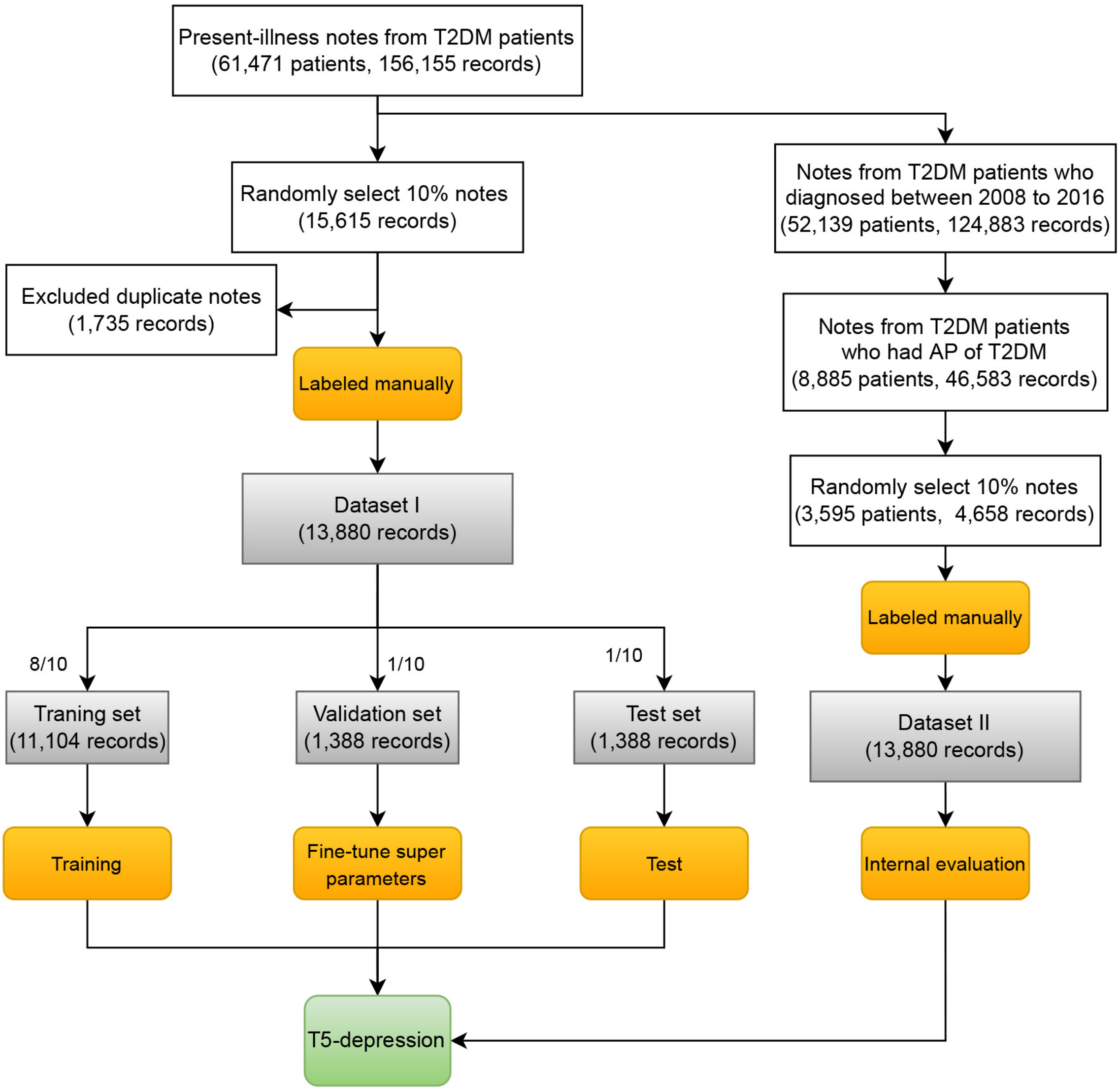

Figure S2. Flowchart of record selection and training, validation, testing, and internal evaluation of the T5-depression model.

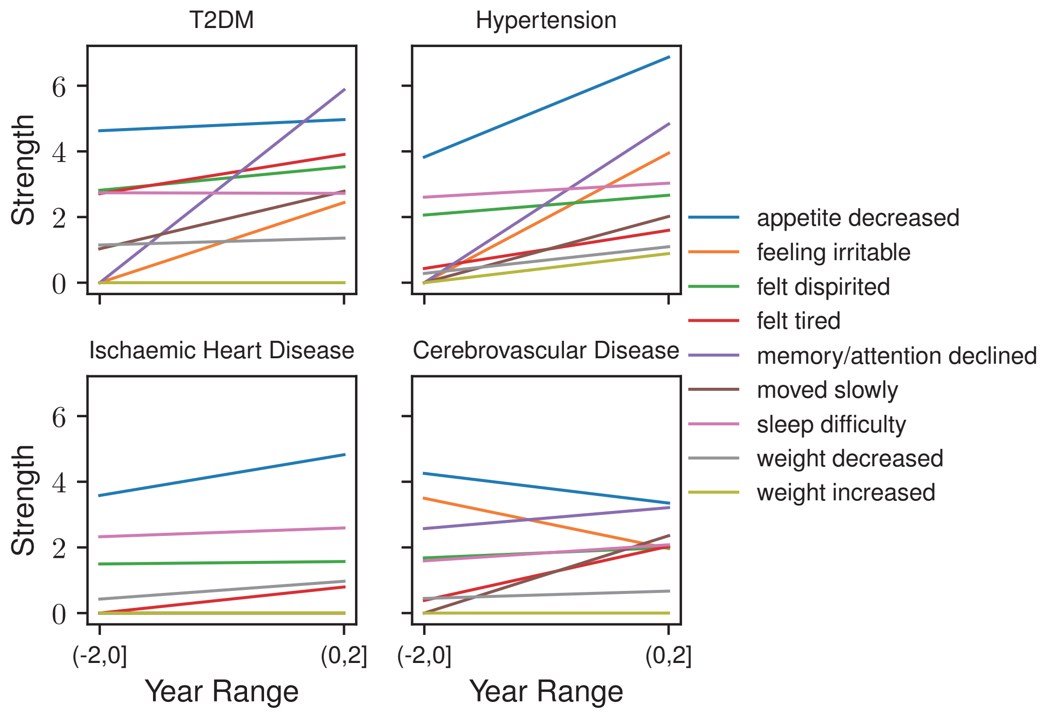

Figure S3. Centrality of each depressive symptoms in networks. (-2, 0], from 2 years before diagnosis date to the diagnosis date of each disease; (0, 2], from diagnosis date to 2 years after the diagnosis date of each disease.

1.3 Tables

Table S1. Year-wise diagnosis dates for eligible patients with T2DM and complications

	2009	2010	2011	2012	2013	2014	2015	2016	2017
Age, mean (SD)	63.23 (15.95)	63.44 (15.3)	61.35 (16.81)	58.88 (16.04)	60.71 (16.1)	59.84 (15.54)	59.75 (15.16)	60.0 (14.35)	
T2DM	329	533	792	1050	1479	1502	1552	1648	
depression	8	20	35	34	49	47	47	58	
Hypertension	26	99	164	286	327	357	467	761	132
IHD	42	81	137	147	185	172	213	311	69
CVD	50	109	160	188	239	248	298	343	58

Abbreviations: T2DM, type 2 diabetes mellitus; IHD, ischemic heart disease; CVD, cerebrovascular disease; SD, standard deviation.

Table S2. Depressive symptoms according to the screen tool and research.

	Items in Questionaire	Symptoms summarized
PHQ-9(Kroenke et al., 2001)	Little interest or pleasure in doing things	Felt dispirited
	Feeling down, depressed, or hopeless	Depressed or anxiety
	Trouble falling or staying asleep, or sleeping too much	Sleep difficulty
	Feeling tired or having little energy	Felt tired
	Poor appetite or overeating	Appetite decreased
	Feeling bad about yourself - or that you are a failure or have let yourself or your family down	Felt dispirited
	Trouble concentrating on things, such as reading the newspaper or watching television	Memory/attention declined
	Moving or speaking so slowly that other people could have noticed? Or the opposite – being so fidgety or restless that you have been moving around a lot more than usual	Moved slowly, feeling irritable
	Thoughts that you would be better off dead or of huring yourself in some way	Suicide attempt
WHO-5(Topp et al., 2015)	... I have felt cheerful and in good spirits	Felt dispirited
	... I have felt calm and relaxed	Feeling irritable
	... I have felt active and vigorous	Felt tired
	... I woke up feeling fresh and rested	Depressed or anxiety
	... my daily life has been filled with things that interest me	Felt dispirited

Table S3. Keywords of depressive symptoms from EHR notes.

Depressive symptoms	Synonyms
Felt tired	General fatigue; feel tired; lack strength; get tired easily;
Sleep difficulty	Unable to sleep; poor sleep quality; difficult to lie flat at night;restless sleep;
Appetite decreased	Anorexia; poor appetite; appetite decreased; eat less;
Moved slowly	Move slowly; difficulty walking;
felt irritable	Feel irritable; capricious moods; get provovation easily;
Memory or attention declined	distracted easily; attention declined; memory declined; memory disorder;
Weight decreased	Weight decreased; emaciation;
Weight increased	Weight increased;
Felt dispirited	Feel dispirited; poor mental state; low spirits;

Table S4. Descriptive statistics for Dataset I, II, and III.

	Dataset I	Dataset II
Total notes (*n*)	13880	4658
Patients (*n*)	9959	3595
Sex (%)		
Male	6182 (56.89)	2207 (61.39)
Female	4684 (43.11)	1388 (38.61)
Age, mean (SD)	60.81 (15.68)	71.55 (8.02)
Complications (%)		
Ischaemic Heart Disease	2005 (18.45)	673 (18.72)
Cerebrovascular Disease	2228 (20.50)	815 (22.67)
Hypertension	7027 (64.67)	2589 (72.02)
Depressive Symptoms in notes		
felt tired	3115	751
sleep difficulty	2375	572
appetite decreased	2705	693
memory/attention declined	412	47
moved slowly	267	74
felt irritable	215	17
weight decreased	1885	383
weight increased	163	23
depressed or anxiety	62	8
suicide attempt	0	0
felt dispirited	971	166

For age, standard deviation (SD) is given in parentheses, mean value is outside. For sex and complications, percentage value is given in parentheses, number of patients is outside.

Table S5. Annotation model metrics for different symptoms in Dataset I.

	Rule-based			Bert			Roberta			Text2event		
Depressive Symptoms	P	R	F1	P	R	F1	P	R	F1	P	R	F1
Felt tired	99.34	92.31	95.69	93.92	91.69	92.97	93.67	92.28	92.97	93.92	95.08	94.50
Sleep difficulty	95.19	75.74	84.36	93.64	80.16	86.37	92.27	83.66	87.76	89.68	96.17	92.81
Appetite decreased	90.40	65.57	76.01	89.70	80.38	84.79	86.57	89.23	87.88	89.24	94.14	91.62
Moved slowly	94.74	75.00	83.72	70.59	57.14	63.16	62.50	71.43	66.67	66.67	75.00	70.59
Felt irritable	100	73.68	84.85	76.92	43.48	55.56	90.48	82.61	86.36	72.22	68.42	70.27
Memory or attention declined	100	94.74	97.30	97.44	95.00	96.20	92.68	95.00	93.83	92.68	100.00	96.20
Weight decreased	90.57	75.39	82.29	91.12	78.57	84.38	89.73	84.69	87.14	87.50	91.62	89.51
Weight increased	85.71	35.29	50.00	80.00	80.00	80.00	82.35	93.33	87.50	92.86	76.47	83.87
Felt dispirited	100	92.45	96.08	97.70	86.73	91.86	97.70	86.73	91.89	90.99	95.28	93.09
Micro-average	**95.46**	79.85	86.96	92.41	83.04	87.47	90.65	87.81	89.21	89.81	**93.89**	**91.80**

Abbreviations: P, Precision; R, Recall; F1, F-score;
Micro-averaging was performed on all types of symptoms by counting the total true positives, false negatives, and false positives.

REFERENCES

Kroenke K, Spitzer RL, Williams JB. The PHQ-9: validity of a brief depression severity measure. *Journal of General Internal Medicine* **16** (2001) 606–613. doi:10.1046/j.1525-1497.2001.016009606.x.

Topp CW, Østergaard SD, Søndergaard S, Bech P. The WHO-5 Well-Being Index: A Systematic Review of the Literature. *Psychotherapy and Psychosomatics* **84** (2015) 167–176. doi:10.1159/000376585.
